# Assessing NH300094, a novel dopamine and serotonin receptor modulator with cognitive enhancement property for treating schizophrenia

**DOI:** 10.3389/fphar.2024.1298061

**Published:** 2024-01-24

**Authors:** Zijin Feng, Zhijing Hu, Lei Li, Minquan Yu, Yiting Zhang, Peng Jing, Xiangqing Xu, Jinhui Wu, Yiqiao Hu, Xiangyang Xu

**Affiliations:** ^1^ State Key Laboratory of Pharmaceutical Biotechnology, Medical School, Nanjing University, Nanjing, China; ^2^ Shanghai Shujing Biopharma Co., Ltd., Shanghai, China; ^3^ Jiangsu Nhwa Pharmaceutical Co., Ltd. & Jiangsu Key Laboratory of Central Nervous System Drug Research and Development, Xuzhou, China

**Keywords:** schizophrenia, antipsychotic, dopamine and serotonin receptors, cognitive improvement, risperidone

## Abstract

**Background:** Schizophrenia is a serious psychiatric disorder that significantly affects the quality of life of patients. The objective of this study is to discover a novel antipsychotic candidate with highly antagonistic activity against both serotonin and dopamine receptors, demonstrating robust efficacy in animal models of positive, negative, and cognitive symptoms of schizophrenia.

**Methods:** In the present study, we examined the activity of antipsychotic drug (NH300094) on 5-HT_2A_, 5-HT_2C_, 5-HT_1A_, 5-HT_1B_, 5-HT_7_, H_1_, M_1_, Alpha_1A_, D_2L_, D_2S_, Alpha_2A_, D_3_ receptor functional assay *in vitro*. In addition, multiple animal models, including dizocilpine (MK-801) induced hyper-locomotion; APO induced climbing; Conditioned Avoidance Response (CAR); DOI-Induced Head Twitch; Forced swimming test; Scopolamine induced cognitive impairment model, were used to verify the antipsychotic activity of NH300094 in preclinical.

**Results:** *In vitro* functional assays have indicated that NH300094 is a potent antagonist of 5-HT receptors and dopamine receptors, with higher relative antagonistic activity against 5-HT_2A_ receptor (5-HT_2A_ IC_50_ = 0.47 nM) than dopamine receptors (D_2L_ IC_50_ = 1.04 nM; D_2S_ IC_50_ = 11.71 nM; D_3_ IC_50_ = 31.55 nM). Preclinical *in vivo* pharmacological study results showed that NH300094 was effective in multiple models, which is more extensive than the clinic drug Risperidone. Furthermore, the safety window for extrapyramidal side effects of NH300094 is significantly wider than that of Risperidone (For NH300094, mice catalepsy model ED_50_/ Mice MK-801 model ED_50_ = 104.6-fold; for Risperidone, mice catalepsy model ED_50_/ Mice MK-801 model ED_50_ = 12.9-fold), which suggests a potentially better clinical safety profile for NH300094.

**Conclusion:** NH300094 is a novel potent serotonin and dopamine receptors modulator, which has good safety profile and therapeutic potential for the treatment of schizophrenia with cognition disorders.

## 1 Introduction

Schizophrenia is a serious psychiatric disorder that significantly affects the quality of life of patients. It has a variety of psychopathological features including positive symptoms (hallucinations and delusions), negative symptoms (social withdrawal, spontaneous speech reduction, impaired motivation), and neurocognitive disorders (Owen et al., 2016). The global incidence of schizophrenia is about 1%, and the lifetime prevalence of patients is 0.7%–0.8% (Schultz et al., 2007; Lohrs and Hasan, 2019). It is estimated that there are more than 21 million schizophrenics worldwide (Kane et al., 2019). Antipsychotics are still the main treatment for schizophrenia. Typical and atypical antipsychotic drugs are two main categories of clinical drugs to control schizophrenia. However, these current drugs have many side effects and can only control part of the symptoms of patients. For example, typical antipsychotic drugs have limited efficacy in treating negative symptoms of schizophrenia, but cause extrapyramidal reactions (EPS), tardive dyskinesia and other adverse effects. Atypical antipsychotic drugs, e.g., risperidone, can prolonged the time interval from the beginning of the QRS complex to the end of the T-wave (QT interval), prolactin elevation and other adverse effects (Bhana et al., 2001; Lin et al., 2010; Adams et al., 2013). Additionally, current antipsychotic drugs are ineffective in about 30% of patients with treatment-resistant schizophrenia (Kane et al., 2019; Chakrabarti, 2021). Clozapine is the only drug currently recommended for refractory schizophrenia, but it is susceptible to obesity and fatal agranulocytosis, which limit its clinical use (Mijovic and MacCabe, 2020). Therefore, there are significant unmet medical needs for new antipsychotic drugs with more efficacious but less side effects (Kantrowitz et al., 2023).

Modulation of serotonin and dopamine receptors in the central nervous system has proven to be an effective way to treat psychiatric disorders (Malik et al., 2023; Stelmach et al., 2023). The dopamine receptor is the crucial target of all existing antipsychotics. Dopamine D_2_ receptor antagonists are thought to control positive symptoms in patients with schizophrenia (Farde et al., 1988; Casey and Canal, 2017; Juza et al., 2022). Schizophrenia may be controlled by the antagonism of the 5-HT_2A_ receptor in synergy with the antagonism of the dopamine D_2_ receptor (Andree et al., 1997). However, many atypical antipsychotics have more selectivity for dopamine D_2_ receptor than 5-HT_2A_ receptor at therapeutic doses in clinical. These atypical antipsychotic drugs with relatively high D2 receptor occupancy in the striatum and presumably other D2 expressing tissues such as pituitary gland, elevate prolactin levels and can induce extrapyramidal motor side effects at therapeutic doses. Selective 5-HT_2A_ receptor antagonist has been proved not only to enhance dopamine D2 receptor antagonist-mediated antipsychotic efficacy but also to reduce hyperprolactinemia and motor side effects (Wadenberg et al., 2001; Gardell et al., 2007).

Furthermore, the dopamine D_3_ receptor may represent an important target for antipsychotic drugs (Schwartz et al., 2000; Gross et al., 2013). The dopamine D_3_ receptor has been recognized to have several central nervous system (CNS) functions, such as social behavior, movement control, emotional regulation, reward, learning, and cognition function (Kiss et al., 2021). Dopamine D3 receptor antagonists possess improving cognitive impairment activity, which may benefit the treatment of cognitive dysfunction associated with several psychiatric disorders (Laszy et al., 2005). Therefore, a medication that combines potent 5-HT_2A_ receptor antagonism with optimal dopamine D_2_ receptor modulation, and the dopamine D_3_ receptor antagonism activity may present an ideal balance of dopaminergic and serotonergic neurotransmitter for the treatment of schizophrenia.

Taken together, we proposed the hypothesis that compounds acting synergistically on serotonin and dopamine receptors might be able to address schizophrenic symptoms with less or without inducing extrapyramidal symptoms (EPS) and other side effects. A series of fused heterocyclic derivatives were synthesized, which have potent activity in serotonin and dopamine receptors. The patent for the synthesis of the compounds has been published (Jing et al., 2021). NH300094 (8-(3-(4-(6-fluorobenzo [d]isoxazol-3-yl) piperidin-1-yl) propoxy)-1,2,5,6-tetrahydro-4H-pyrrolo [3,2,1-ij] quinolin-4-one) was characterized as a preclinical candidate compound based on the good preclinical profiles, which is a triple antagonist of 5-HT_2A_ receptor, dopamine D_2_ receptor and dopamine D_3_ receptor. Additionally, it has strong inverse agonist activity of 5-HT_1B_ and antagonistic activity of 5-HT_1A_ receptor. NH300094 is being developed and clinically intended for the treatment of positive symptoms, negative symptoms and cognitive disorders of schizophrenia.

## 2 Materials and methods

### 2.1 Experiment cells

CHO-K1/5-HT_2A_ and CHO-K1/D_3_ cells were purchased from Shanghai PerkinElmer Biotechnology Co., Ltd. CHO-K1/M_1_, HEK293/H_1_, CHO-K1/D_2_, HEK293/5-HT_7_ and CHO-K1/5-HT_1A_ cells were purchased from Nanjing GenScript Biotechnology Co., Ltd. HEK293/5-HT_2C_, HEK293/Alpha_1A_ and HEK293/Alpha_2A_ cells were constructed by biology laboratory of Shanghai shujing Biopharma Co., Ltd. CHO-K1/5-HT_1B_ cells were purchased from Wuhan Creater Biotechnology Co., Ltd.

### 2.2 Experimental animals

Male Wistar rats (weight, 200–230 g) and ICR mice (weight, 20–28 g) were purchased from SPF (Beijing) Biotechnology Co., Ltd. Male Sprague-Dawley (SD) rats (weight, 180–220 g) were purchased from Beijing Vital River Laboratory Animal Technology Co., Ltd. The animals were group feeding under standard conditions (temperature:20°C–26°C, humidity: 40%–70%, 12-h dark/light cycle). Before the testing, the animals were acclimated to the laboratory environment for 3 days, and the animal food and drinking water were freely provided. The animal experiment protocols were approved by the Institutional Animal Care and Use Committee of Jiangsu Nhwa Pharmaceutical Co., Ltd.

### 2.3 Drugs

**FIGURE 1:**
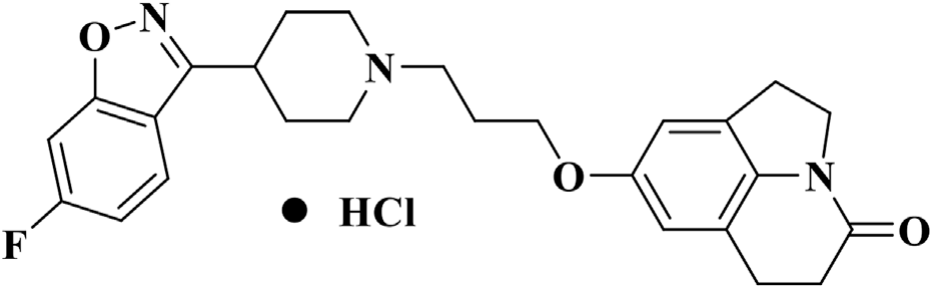
Chemical structure of NH300094.

NH300094 hydrochloride, risperidone and duloxetine hydrochloride were synthesized at Jiangsu Nhwa Pharmaceutical Co., Ltd. The molecular structure of NH300094 (PCT/CN 2020/129850) is shown in Figure 1. Other compounds such as (+)-MK-801 hydrogen maleate (M107-250MG, Sigma), rivastigmine hydrogen tartrate (LRAB1259, Sigma), R (2)-2,5-dimethoxy-4-iodoamphetamine (DOI) (D101-100MG, Sigma), R-(−)-Apomorphine (APO) hydrochloride (A4393, Sigma), L-Ascorbic acid Vc (A5960-25G, Sigma), (+)-Butaclamol (D033, Sigma) were purchased from Sigma-Aldrich (St. Louis, MO). Scopolamine hydrobromide (S107418-5g, Aladdin) was purchased from Aladdin. WAY-00635 (T2631, Targetmol), Ketanserin (T1066, Targetmol), Yohimbine (T2142, Targetmol), Pyrilamine (T1232, Targetmol), Atropine (T0375, Targetmol), Prazosin (T1050, Targetmol) were purchased from Targetmol. Methiothepin (HY-107836, MCE) was purchased from MCE. All drugs were dissolved in normal saline or deionized water and administered orally at 10 mL/kg (volume/body weight), unless otherwise indicated.

### 2.4 Receptor functional activity

#### 2.4.1 5-HT_2A_, 5-HT_2C_, H_1_, M_1_, Alpha_1A_ receptor functional assay

A calcium flow assay was used to test the activity of compounds on 5-HT_2A_, 5-HT_2C_, H_1_, M_1_, Alpha_1A_ receptor. After the CHO-K1/5-HT_2A_, CHO-K1/M_1_, HEK293/5-HT_2C_, HEK293/H_1_ and HEK293/Alpha_1A_ cells were lightly trypsinized, a density of 2×10^4^ cells/well were inoculated in 384-well plates (Greiner-781946) which containing 20 μL cell medium in each well. The cells were routinely cultured at 37°C with 5% CO_2_ for 16–24 h prior to testing.

In agonist experiments, 20 μL of experimental buffer was added to each well of the assay plate after removing the medium. Then, 20 μL of fluorescent probe solution was added to each well. The assay plate was incubated in incubator at 37°C for 50 min, let it stand at room temperature for 10 min, then transferred to the reading position of the Fluorescent Image Plate Reader (FLIPR). The compound (10 μL) was added to the assay plate and the fluorescence signal was read for 210 s.

For the antagonist tests, 20 μL of experimental buffer and fluorescent probe solution was added to each well of the assay plate after removing of the culture medium. The assay plate was incubated in incubator at 37°C for 50 min, let it stand at room temperature for 10 min, then transferred to the reading position of the Fluorescent Image Plate Reader (FLIPR). The compound and control agonist serotonin (10 μL) was added to the assay plate and the fluorescence signal was read for 210 s. After reading the raw data from FLIPR, the EC_50_ and IC_50_ were calculated, respectively.

For both agonist and antagonist tests, the difference between the maximum value and the minimum value of fluorescence signal readings (rang, 1–210 s) was regarded as the change of relative fluorescent unit intensity (△RFU). The agonistic or antagonistic activity of drug was analyzed using the following equation:

$$\%Activity{=(∆\text{RFU}}_{\text{Compound}}{‐∆\text{RFU}\;}_{\text{negative}\;\text{control}}{)/(∆\text{RFU}\;}_{\text{positive}\;\text{control}}{‐∆\text{RFU}\;}_{\text{negative}\;\text{control}})\times 100$$

$$\%Inhibition=100‐\left({∆\text{RFU}}_{\text{Compound}}{‐∆\text{RFU}\;}_{\text{positive}\;\text{control}}{)/(∆\text{RFU}\;}_{\text{negative}\;\text{control}}{‐∆\text{RFU}\;}_{\text{positive}\;\text{control}}\right)\times 100$$

Dose-response curves for agonist/antagonist were fitted using the software GraphPad Prism (version 8.0.2) with four parameter logistic equation.

#### 2.4.2 D_2L_, D_2S_, 5-HT_1A_, 5-HT_1B_, Alpha_2A_ receptor functional assay

The activity of the compounds on D_2L_, D_2S_, 5-HT_1A_, 5-HT_1B_ and Alpha_2A_ receptors was detected using cAMP assay. The CHO-K1/D_2_, CHO-K1/5-HT_1A_, HEK293/Alpha_2A_ and CHO-K1/5-HT_1B_ cells were diluted to the appropriate concentration with experimental buffer, and 10 μL of the cell solution was transferred to each well of the assay plate. The compound was then transferred to the assay plate using Tecan-D300e. After centrifuging at 1,000 rpm for 1 min, the assay plate was incubated for 15 min at room temperature. The appropriate amount of Forskolin solution was added to the cell plate, where the antagonist test assay requires an additional positive compound (dopamine for D_2L_, D_2S_; serotonin for 5-HT_1A_, 5-HT_1B_; DL-Adrenaline for Alpha_2A_), centrifuged at 1,000 rpm for 1 min. The plate was incubated for 45 min at room temperature before adding 10 μL cAMP-d_2_ solution and anti-cAMP-Cryptate solution to the assay plate, which was then centrifuged at 1,000 rpm for 1 min. The plate was incubated at room temperature for 1 h. And then read using Envision (PerkinElmer) with parameters set to excitation 340 nm and emission 620 nm/665 nm.

#### 2.4.3 D_3_ receptor functional assay

For the D_3_ receptor functional assay, the Nano-Glo^®^ luciferase assay system was used to detect the activity of the compounds. The CHO-K1/D_3_ cells were diluted to a final concentration of 5×10^5^ cells/mL, and 20 μL of cell suspension (cell density of 10,000 cells/well) was added to each well of a 384-cell plate and incubated for 16–24 h in a 5% CO_2_ and 37°C incubator. The compounds were transferred to the assay plate using a Tecan-D300e, centrifuged at 1,000 rpm for 1 min, and incubated at 37°C for 30 min. After adding the Forskolin solution, the assay plate was centrifuged at 1,000 rpm for 1 min and incubated at 37°C for 4 h. The substrate and assay buffer (v/v, 1/50) were added to the assay plate, and then incubated at room temperature for 5 min after centrifuging at 1,000 rpm for 1 min. Finally, the assay plate was read using enzyme-labelling measuring instrument and the IC_50_ of compounds was calculated.

#### 2.4.4 5-HT_7_ receptor functional assay

The activity of the compounds on 5-HT_7_ receptors was detected using Bright-Glo™ Luciferase assay system. The HEK293/5-HT_7_ cells were diluted to the 20000 cells/well with assay buffer, and 20 μL of the cell solution was transferred to each well of the assay plate (384-well plate). The test compound and positive compound were then transferred to the assay plate using Tecan-D300e. After centrifuging at 1,000 rpm for 1 min, the assay plate was incubated for 30 min at 37°C. The serotonin solution was transferred into assay plate, centrifuge at 1,000 rpm for 1 min, and incubated for 4 h at 37°C. After that the 30 μL of detection reagent was added to the cell plate, centrifuge at 1,000 rpm for 1 min, and read using Envision with the HTRF compatible reader.

### 2.5 *In vivo* pharmacological study

#### 2.5.1 MK-801-induced hyperactivity in mice

According to the body weight, one hundred and twenty male ICR mice (5 weeks of age) were randomly divided into ten groups with twelve mice per group. The mice were then dosed with vehicle (*p.o.*), NH300094 (0.1, 0.3, 1 and 3 mg/kg, *p.o.*) or risperidone (0.1, 0.3, 1 and 3 mg/kg, *p.o.*) and placed back into their home-cage for 30 min. Immediately after injection with either saline or MK-801 (0.3 mg/kg, *i.p*.), the mice were placed into the test chambers (29 cm × 29 cm × 30 cm) for 60 min of locomotion recording using a tracking and computerized analysis system (TopScan Version 3.00, Clever Sys Inc., Leesburg, VA). After each test, the test chamber should be cleaned and wiped with 75% alcohol solution. The detailed flow chart of the test method is presented in Supplementary Figure S1A.

#### 2.5.2 APO-induced climbing in mice

According to the body weight, one hundred and eight male ICR mice (5 weeks of age) were randomly divided into nine groups with twelve mice per group. The mice were treated with either vehicle (*p.o.*), NH300094 (0.03, 0.1, 0.3 and 1 mg/kg, *p.o.*) or risperidone (0.03, 0.1, 0.3 and 1 mg/kg, *p.o.*) and placed back into their home-cage for 60 min. Afterward, mice were injected with apomorphine (APO, 1 mg/kg, *s.c.*) and immediately placed individually into cylindrical cages (13 cm diameter, 15 cm high, with walls of vertical bars, 1 cm diameter) for behavior observation. The behavior of the mice was observed and scored at 10–11, 20–21, 30–31 min post injection of APO as follows: 0 = four paws on the cage floor; 1 = two paws holding the vertical bars of the cage; 2 = four paws holding the vertical bars of the cage. After each test, the test chamber should be cleaned and wiped with 75% alcohol solution. The detailed flow chart of the test method is presented in Supplementary Figure S1B.

#### 2.5.3 DOI-induced head twitch in mice

The test referred to the previously description of the DOI-induced head twitch test in mice (Fantegrossi et al., 2010). One hundred and eight male ICR mice (5 weeks of age) were randomly divided into nine groups with twelve mice per group: control, NH300094 (0.001, 0.003, 0.01, and 0.03 mg/kg, *p.o.*), risperidone (0.001, 0.003, 0.01 and 0.03 mg/kg, *p.o*.). The mice were administered intragastrically with vehicle or compounds 60 min before the DOI (1 mg/kg, *i.p.*) injection. After that, the mice were immediately placed into the plexiglass box individually. The number of head twitches in the mice was counted by the blind observer over a 20-min period. The detailed flow chart of the test method is presented in Supplementary Figure S1C.

#### 2.5.4 Conditioned avoidance response test in rats

The experiments were conducted in two phases: Phase I, conditioned avoidance response (CAR) training (112 male Wistar rats aged 7 weeks which were used when study started); Phase II, grouping the qualified rats and testing the efficacy of compounds in CAR test. Shuttle-box Avoidance Test Video Analysis System (DigBehv-SBG, Shanghai Jiliang Software Technology Co. Ltd.) were used to assess the rats conditioned avoidance response.

Phase I: The rats responded to the conditioned stimulus (auditory and visual) by training with foot shock reinforcement. Briefly, rats were placed into the CAR shuttle boxes for a 5-min habituation followed by 30 trials presented on a 20-s variable interval (20–30 s) stimulus. Each rats were subjected to a conditioned stimulus which consisted of 10s presentation of light and white noise, and then followed by a scrambled 1.5 mA foot shock for 10 s. Rats were recorded as “avoidance” if they had successfully moved to the other compartment during the stimulus process; Rats that ran to the other compartment during the shock was recorded as “escape”; Rats that failed to move to the other compartment during the shock period were recorded as “escape failure”. Rats with avoidance rates greater than 70% for 3 consecutive days were included in this study post-training.

Phase II: The qualified rats were randomly divided into 7 groups. Each rats were individually placed into a shuttle box for CAR testing 1 h post oral administration of vehicle, NH300094 (0.3, 1 and 3 mg/kg, *p.o.*) or risperidone (0.3, 1 and 3 mg/kg, *p.o.*). The procedure in the testing phase was the same as the Phase I described. The number of avoidances, escapes, and escape failures were recorded. The detailed flow chart of the test method is presented in Supplementary Figure S1D.

#### 2.5.5 Novel object recognition in mice

The procedure was modified according to Bevins and Besheer (Bevins and Besheer, 2006). According to the body weight, one hundred and sixty male ICR mice (5 weeks of age) were randomly divided into ten groups with sixteen mice per group. The tests were conducted in a 50 cm × 35 cm × 20 cm chamber and the mice behavior was recording using a Hikvision video recording system (H.265, Hikvision Digtial Technology Co., Ltd.). All mice were allowed to freely explore the chamber environment for 10 min, and there were no objects placed in the chamber during acclimatization period. About 24 h after habituation, mice were dosed with vehicle (*p.o.*), NH300094 (0.04, 0.08 and 0.16 mg/kg, *p.o.*) or risperidone (0.04, 0.08 and 0.16 mg/kg, *p.o.*) 30 min before injected with scopolamine hydrobromide (3 mg/kg, *i.p.*). Rivastigmine hydrogen tartrate group (0.1, 0.3 and 1 mg/kg, *i.p.*) were dosed simultaneously with scopolamine hydrobromide (3 mg/kg, *i.p.*). Training was conducted 30 min post scopolamine hydrobromide administration by placing a single mouse into a chamber for 10 min with two exactly same objects positioned in the center of chamber. (The distance between the two objects was more than 20 cm). The short-term memory of mice was tested 1 h post training by exploring the chamber for 10 min in the presence of a new and familiar object. The 10-min testing was videotaped. After each test, the test chamber should be cleaned and wiped with 75% alcohol solution. All of the objects presented had similar sizes, colors and textures.

The experimental video was analyzed to record the time of mice exploring the new and old objects, respectively, and the differentiation index (DI) was calculated as follows: DI = new object exploration time/(new object exploration time + old object exploration time), which was used as the main evaluation index of discrimination ability. Analysis stopped when the total exploration time of the new and old objects reaches 20 s. If the total exploration time of the new and old objects was less than 20 s, the total 10 min of video would be analyzed. Exploration was defined as the distance between the nose of mouse and the object being less than 1 cm when the mouse actively explored the object. The movement of circling without sniffing or sitting on the object was not recorded as exploration behavior. The detailed flow chart of the test method is presented in Supplementary Figure S1E.

#### 2.5.6 Forced swimming test in mice

The test referred to the previously description of the forced swimming test (FST) in mice (Porsolt et al., 1978). One hundred and sixty male ICR mice (5 weeks of age) were randomly divided into ten groups with sixteen mice per group: vehicle control, duloxetine (20 mg/kg, p.o.), risperidone (0.01, 0.03 and 0.1 mg/kg, p.o.) and NH300094 (0.003, 0.01 and 0.03 mg/kg, p.o.), the dosage of duloxetine was referred to the previous study (Xu et al., 2018). Each mouse was required to swim in an open cylindrical container (height of 25 cm, diameter of 10 cm) after intragastric administration of compounds 1 h. The container contained 1.2 L of water with temperature maintained at 24°C ± 1°C. The test used a computerized analysis and tracking system (Clever Sys Inc., Leesburg, VA) to recorded the duration of immobility (last 4 min of a total time of 6 min) about the testing mice. The detailed flow chart of the test method is presented in Supplementary Figure S1F.

#### 2.5.7 Spontaneous locomotor activity test in mice

One hundred and eight male ICR mice (5 weeks of age) were randomly divided into nine groups with twelve mice per group. One hour after oral administration of vehicle (*p.o.*), NH300094 (0.1, 0.3, 1 and 3 mg/kg, *p.o.*), risperidone (0.1, 0.3, 1 and 3 mg/kg, *p.o.*), mice were individually placed into test chamber (29 cm × 29 cm × 30 cm) for locomotion recording for 60 min using a computerized analysis and tracking system (Clever Sys Inc., Leesburg, VA). After each test, the test chamber should be cleaned and wiped with 75% alcohol solution. The detailed flow chart of the test method is presented in Supplementary Figure S1G.

#### 2.5.8 Catalepsy test in mice

The test referred to the previously description of the catalepsy test in mice (Kuschinsky and Hornykiewicz, 1972). According to the body weight, one hundred and eight male ICR mice (5 weeks of age) were randomly divided into nine groups with twelve mice per group. Catalepsy was assessed at 30, 60 and 90 min post oral administration of vehicle (*p.o.*), NH300094 (1, 3, 10 and 30 mg/kg, *p.o.*) or risperidone (0.1, 0.3, 1 and 3 mg/kg, *p.o.*). The front paws of mice were placed on a horizontal stainless bar (length: 20 cm; diameter: 0.3 cm; height: 5.5 cm). If this behavior of mouse lasted for 30 s or longer, catalepsy would be considered as positive, and 60 s was used as cut-off. After each test, the test area should be cleaned and wiped with 75% alcohol solution. The detailed flow chart of the test method is presented in Supplementary Figure S1H.

### 2.6 Pharmacokinetics assay

Six male SD rats (7 weeks of age) were randomly divided into two groups with three rats per group. Animals were fasted overnight and had free access to water before dosing. For the intravenous group, male SD rats were administered NH300094 by single intravenous bolus administration at a dose of 2 mg/kg. For the oral group, male SD rats were dosed orally with NH300094 at a dose of 10 mg/kg. Serial blood samples were collected at different time points (Pre-dose, 0.033, 0.083, 0.25, 0.5, 1, 2, 4, 6, 8, 12 and 24 h for the intravenous group; Pre-dose, 0.167, 0.333, 0.5, 1, 2, 4, 6, 8, 10, 12 and 24 h for the oral group) via jugular vein puncture from each study animal. All blood samples were transferred into commercial tube containing K_2_-EDTA. Plasma samples were prepared by centrifuging the blood samples at approximately 4°C, 3,200×g for 15 min, and then stored at −70°C until analysis (Supplementary Materials). The pharmacokinetic parameters were calculated using WinNonlin software (Version 6.3) according to non-compartmental model.

### 2.7 Statistical analysis

All raw data were calculated as the mean ± standard deviation (S.D.). Statistical analyses were conducted using GraphPad Prism version 8.0.2 (GraphPad Software). For *in vitro* assays, the IC_50_ and EC_50_ values were calculated by nonlinear regression analysis. For *in vivo* experiments, the data were analyzed statistically by one-way ANOVA followed by Dunnett’s multiple comparison test (*p* < 0.05).

## 3 Results

### 3.1 *In vitro* pharmacology

**FIGURE 2:**
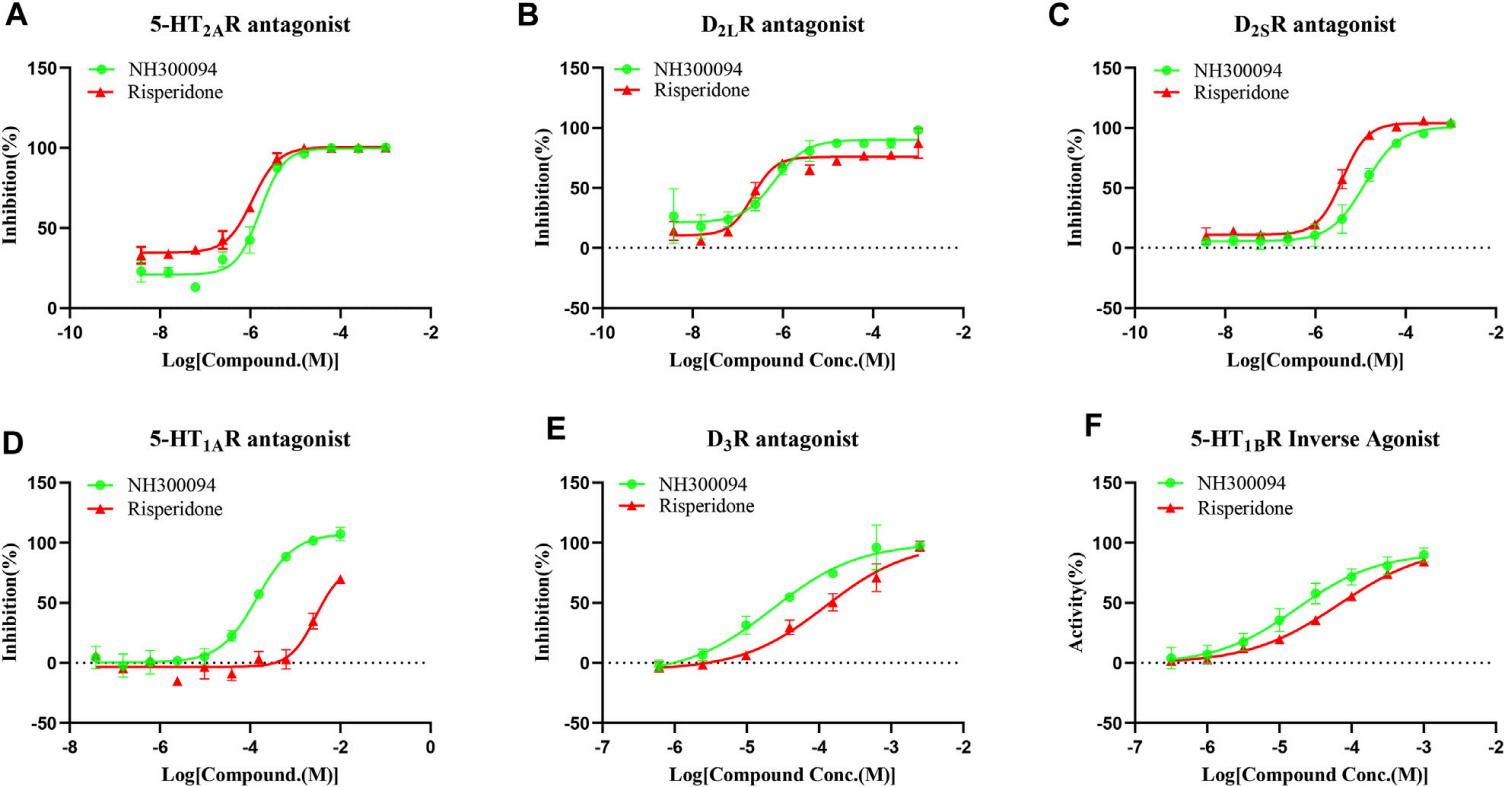
The *in vitro* pharmacological activity of NH300094 and risperidone at 5-HT_2A_R **(A)**, D_2L_R **(B)**, D_2S_R **(C)**, 5-HT_1A_R **(D)**, D_3_R **(E)**, 5-HT_1B_R **(F)**, which presented using concentration-dependence curves. The effects are determined by cAMP production or intracellular Ca^2+^ concentrations. Data are presented as means ± S.D. of three independent test.

The results of tests *in vitro* showed that NH300094 has pharmacological activity against various targets, including antagonist activity against D_2L_R, D_2S_R, D_3_R, 5-HT_1A_R and 5-HT_2A_R. Additionally, inverse agonist activity was observed at 5-HT_1B_R (Table 1; Figure 2). The antagonistic activity to D_2L_R (IC_50_ = 1.04 ± 0.59 nM) and 5-HT_2A_R (IC_50_ = 0.47 ± 0.79 nM) was the most significant activity of NH300094, which indicates its potential anti-schizophrenia activity. NH300094 had lower antagonistic activities ratio of 5-HT_2A_R and D_2_R than risperidone (IC_50_ ratio = 0.45 for NH300094, IC_50_ ratio = 1.0 for risperidone), which is speculated lower extrapyramidal side effects (Meltzer et al., 1989). The high inverse agonist activity of 5-HT_1B_R (EC_50_ = 28.36 ± 12.52 nM) and antagonistic activities of 5-HT_1A_R (IC_50_ = 85.59 ± 61.53 nM) and D_3_R (IC_50_ = 31.55 ± 23.08 nM) suggesting that NH300094 has potential to improve not only the positive symptoms but also the cognitive dysfunctions (Laszy et al., 2005; Meneses, 2007; Ohno, 2011). NH300094 has no significant pharmacological activity against other targets in the study (Supplementary Table S1).

**TABLE 1:** *In vitro* functional profile of NH300094 and Risperidone.

Receptor	NH300094	Risperidone	Positive control^a^
D_2L_R, IC_50_(nM)	1.04 ± 0.59	0.44 ± 0.29	1.08 ± 0.88
D_2S_R, IC_50_(nM)	11.71 ± 9.38	2.11 ± 1.08	1.12 ± 1.63
D_3_R, IC_50_(nM)	31.55 ± 23.08	84.63 ± 36.40	70.67 ± 42.69
5HT_1A_R, IC_50_(nM)	85.59 ± 61.53	4964.67 ± 2981.62	0.41 ± 0.28
5HT_1B_R, EC_50_ (nM)	28.36 ± 12.52	371.50 ± 271.30	22.27 ± 9.69
5HT_2A_R, IC_50_(nM)	0.47 ± 0.79	0.44 ± 0.53	1.05 ± 1.01

IC_50_, half maximal inhibitory concentration; EC_50_, half maximal effective concentration; R, receptor.

^a^ Positive control: (+)-Butaclamol (D_2L_R/D_2S_R/D_3_R), WAY-100635 (5HT_1A_R), Methiothepin (5HT_1B_R), Ketanserin (5HT_2A_R).

### 3.2 Effects of NH300094 on MK-801-induced hyperactivity in mice

**FIGURE 3:**
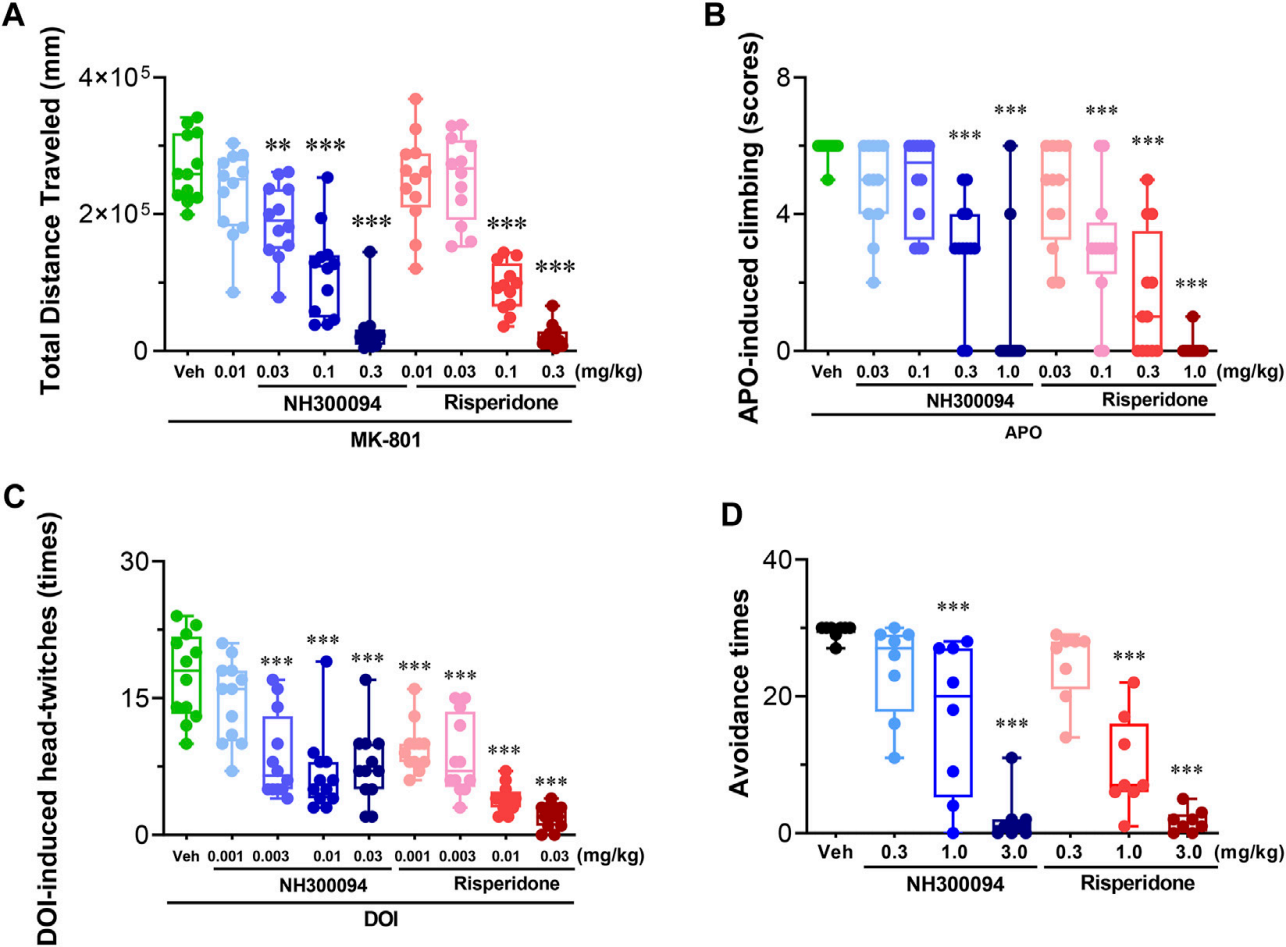
Effects of different doses of NH300094 on schizophrenia-like model compared with antipsychotic drugs risperidone in rodents. **(A)** Effects of NH300094 and risperidone on hyperactivity model induced by MK-801 (0.3 mg/kg, i.p.) in mice (*n* = 12). **(B)** Effects of NH300094 and risperidone on climbing behavior induced by APO (1.0 mg/kg, s.c.) in mice (*n* = 12). **(C)** Effects of NH300094 and risperidone on head-twitches behavior induced by DOI (1.0 mg/kg, s.c.) in mice (*n* = 12). **(D)** Effects of NH300094 and risperidone on the avoidance time of rats in conditional avoidance test (*n* = 8). Data are presented as box-and-whisker plot (min to max with all points) and are analyzed one-way ANOVA with Dunnett’s multiple comparisons tests. **p < 0.01, ***p < 0.001 compared with veh group. Veh: Vehicle.

Single oral dose administrations of NH300094 (0.01, 0.03, 0.1 and 0.3 mg/kg) resulted in a dose-dependent inhibition of MK-801-induced hyperactivity in male ICR mice with an ED_50_ of approximately 0.07 mg/kg and Minimum effective dose (MED) of 0.03 mg/kg. Risperidone (0.01, 0.03, 0.1 and 0.3 mg/kg) also significantly reduced MK-801-induced hyperactivity with an ED_50_ of approximately 0.08 mg/kg and MED of 0.1 mg/kg (Figure 3A).

### 3.3 Effects of NH300094 on APO-induced climbing in mice

Single oral dose administrations of NH300094 (0.03, 0.1, 0.3 and 1 mg/kg) resulted in a dose-dependent inhibition of APO-induced climbing in male ICR mice with an ED_50_ of approximately 0.29 mg/kg and MED of 0.3 mg/kg. Risperidone (0.03, 0.1, 0.3 and 1 mg/kg) also significantly reduced APO-induced climbing behavior, with an ED_50_ of approximately 0.1 mg/kg and MED of 0.1 mg/kg. These results indicate that NH300094 has potential antipsychotic effects in clinic (Figure 3B).

### 3.4 Effects of NH300094 on DOI-induced head twitch in mice

Single oral dose administrations of NH300094 (0.001, 0.003, 0.01 and 0.03 mg/kg) resulted in a dose-dependent inhibition of DOI-induced head twitch in male ICR mice with an ED_50_ of approximately 0.007 mg/kg and MED of 0.003 mg/kg. Risperidone (0.001, 0.003, 0.01, 0.03 mg/kg) also significantly reduced DOI-induced head twitch with an ED_50_ of approximately 0.002 mg/kg and MED of 0.001 mg/kg. The data from this study reveal that both NH300094 has potential antipsychotic effects in clinic. (Figure 3C).

### 3.5 Effects of NH300094 on conditioned avoidance response

Single oral dose administrations of NH300094 (0.3, 1 and 3 mg/kg) resulted in a dose-dependent inhibition of conditioned avoidance response of rats with an ED_50_ of approximately 1.02 mg/kg. Risperidone (0.3, 1 and 3 mg/kg) also significantly reduced conditioned avoidance response of rats with an ED_50_ of approximately 0.70 mg/kg. The results showed that NH300094 has potential antipsychotic effects in clinic (Figure 3D).

### 3.6 Effects of NH300094 on novel object recognition in mice

**FIGURE 4:**
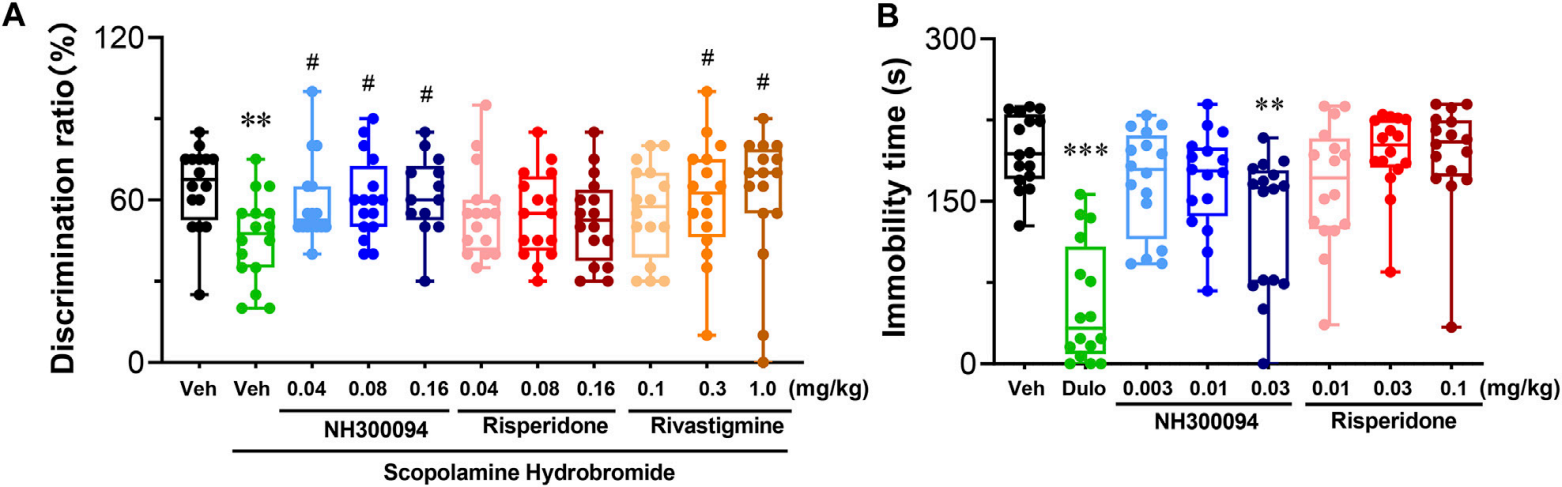
Effects of different doses of NH300094 on cognitive memory and depression-like behavior compared with antipsychotic drugs risperidone in mice. **(A)** The influence of different doses of NH300094 on the discrimination ratio compared to model group (*n* = 16). **(B)** The influence of NH300094 on mobility in FST (*n* = 16). Data are presented as box-and-whisker plot (min to max with all points) and are analyzed one-way ANOVA with Dunnett’s multiple comparisons tests. **p < 0.01, ***p < 0.001 compared with veh group. #*p* < 0.05 compared with model group. Veh: Vehicle.

The cognitive deficits model of mice treated by scopolamine hydrobromide was established, and the effects of NH300094 on the model was examined using novel object recognition (NOR). In the 10-min test experiment, mice dosed with vehicle spent 12.94 s on a novel object and 7.06 s on a familiar object, with a differentiation index (DI) of 0.65. Mice treated with scopolamine hydrobromide showed significant new object recognition impairment by spending roughly equal time exploring an acquainted object and a novel object with a DI of 0.46. Compared with the DI of scopolamine hydrobromide-treated mice, the positive control, rivastigmine bitartrate-treated (0.3 and 1 mg/kg, i.p.) mice also significantly enhanced learning and memory ability, indicating that the testing system worked well for compound testing. In this study, we found that at doses of 0.01–0.16 mg/kg, NH300094 increased DI index of scopolamine hydrobromide-treated mice, while risperidone (0.04, 0.08 and 0.16 mg/kg, p.o.) did not affect the DI of scopolamine hydrobromide-treated mice. These results suggest that NH300094 may have the potential to improve cognitive deficits of schizophrenia (Figure 4A).

### 3.7 Effects of NH300094 on forced swimming test in mice

A single oral dose of administrations of NH300094 (0.03 mg/kg) decreased immobility time in the FST, but single oral dose of administrations of risperidone (0.01, 0.03 and 0.1 mg/kg) did not affect the immobility time in the FST. The results indicate that NH300094 may have potential effects in improving the negative symptoms of schizophrenia, which is different from risperidone (Figure 4B).

### 3.8 Effects of NH300094 on spontaneous locomotor activity

**FIGURE 5:**
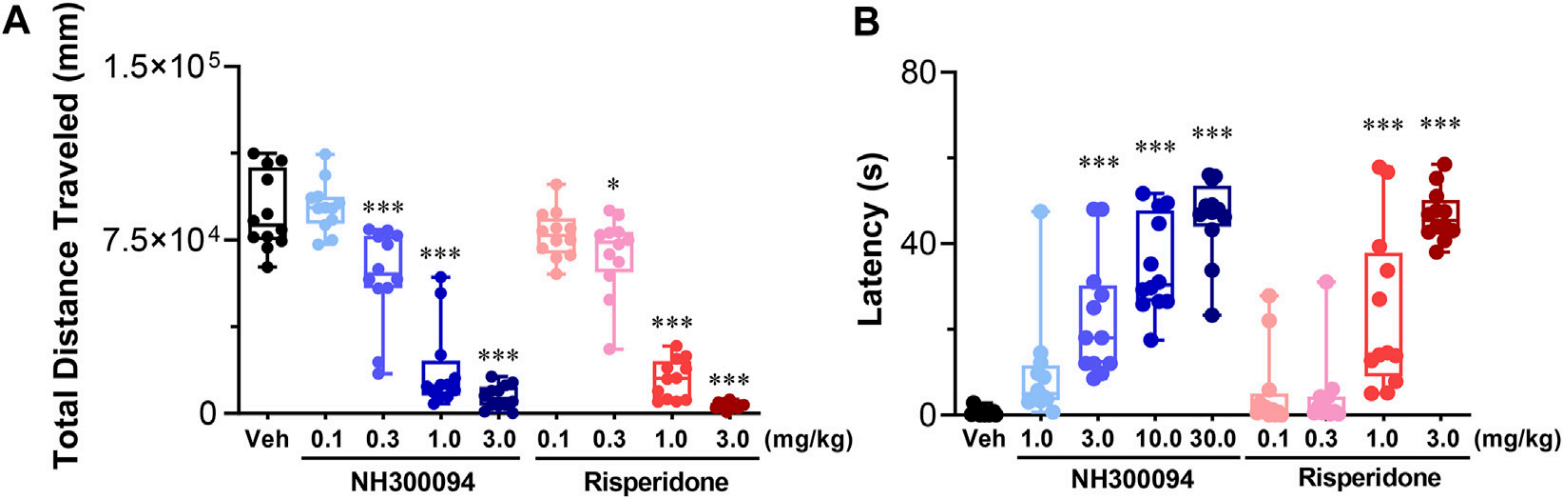
Side-effects of different doses of NH300094 compared with antipsychotic drugs risperidone in mice. **(A)** The influence of single acute treatment with NH300094 and risperidone on the locomotion activity in mice. (n = 12). **(B)** The influence of single acute treatment with NH300094 and risperidone on the catalepsy time in mice. (*n* = 12). Data are presented as box-and-whisker plot (min to max with all points) and are analyzed one-way ANOVA with Dunnett’s multiple comparisons tests. *p < 0.05, **p < 0.01, ***p < 0.001 compared with veh group. Veh: Vehicle.

A single oral dose of administrations of NH300094 (0.1, 0.3, 1 and 3 mg/kg) resulted in a dose-dependent inhibition of spontaneous locomotor activity in male ICR mice with an ED_50_ of approximately 0.49 mg/kg. Risperidone (0.1, 0.3, 1 and 3 mg/kg) also reduced spontaneous locomotor activity significantly with an ED_50_ of approximately 0.52 mg/kg. The ED_50_ of NH300094 in spontaneous locomotor activity is much higher than that of MK-801 induced hyper-locomotor activity, indicating the good safety margin of NH300094 (Figure 5A).

### 3.9 Effects of NH300094 on catalepsy test

The catalepsy test is widely used for evaluating extrapyramidal side effects of dopamine antagonists (Adams et al., 2013). In this study, the minimal dose of NH300094 that induced catalepsy in mice is 3 mg/kg with an ED_50_ of 6.73 mg/kg. On the other hand, the minimal dose of risperidone that induced catalepsy in mice was 1 mg/kg with an ED_50_ of 1.35 mg/kg. These results indicate that NH300094 may have lower EPS side effects compared to risperidone (Figure 5B).

### 3.10 Pharmacokinetics study

**FIGURE 6:**
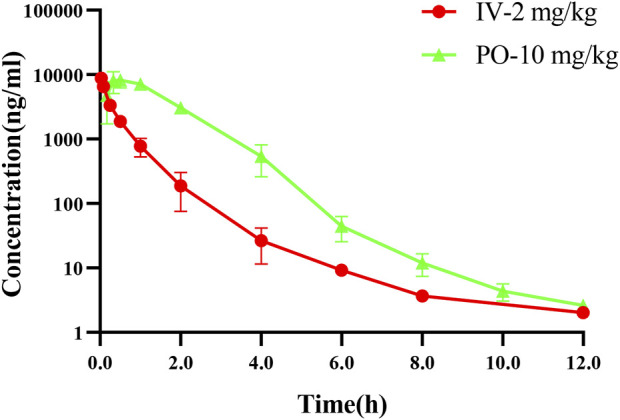
Mean plasma concentration-time profiles of NH300094 in rats after an oral or intravenous administration.

Good pharmacokinetic characteristics are an important factor for clinical efficacy of drugs. The PK parameters of NH300094 were acquired by intravenous and intragastric administration in rats. The mean plasma concentration of NH300094 over 24 h is shown in Figure 6. The oral administration of NH300094 to rats resulted in a half-life of 1.28 h, and the area under the concentration time-curves for time zero to infinity was 14800 ng/mL*h. By comparing the exposure of NH300094 after oral and intravenous administrations, the absolute oral bioavailability of NH300094 was calculated as about 87.6% in rats. The other major pharmacokinetics parameters for different administration routes are presented in Table 2, demonstrating the excellent pharmacokinetic characteristics of NH300094 for further development.

**TABLE 2:** *In vivo* pharmacokinetics parameters of NH300094.

Group	Intravenous administration group	Oral administration group
Administration Dosage (mg/kg)	2	10
C_0_ or C_max_ (ng/mL)	10800	8710
T_max_ (h)	NA	0.389
Cl (mL/min/kg)	9.91	NA
Vd_ss_ (L/kg)	0.379	NA
AUC_0-last_ (ng·h/mL)	3374.4	14782.1
AUC_0-inf_ (ng·h/mL)	3381.4	14787.1
T_1/2_ (h)	1.73	1.28
Bioavailability (%)	NA	87.6

## 4 Discussion

Today, many antipsychotics have been developed, but these drugs have not been able to dissociate efficacy from side effects (Paul et al., 2022; Schneider-Thoma et al., 2022). Additionally, negative symptoms and cognitive dysfunctions of schizophrenia are difficult to manage, which impairs the patient’s ability to return to normal life (Kahn et al., 2015; Xu et al., 2022; Veleva et al., 2023). NH300094 is a novel anti-schizophrenia candidate with unique characteristics of D_2_ receptor, D_3_ receptor antagonism, 5-HT_1A_,5-HT_2A_ receptor antagonism and 5-HT_1B_ inverse agonism. Its antagonistic activity against 5-HT_2A_ receptor is significantly higher than that against the dopamine receptor, which is in line with the characteristics of atypical anti-schizophrenia drugs. Preclinical studies results suggest that NH300094 has the potential to treat positive symptoms of schizophrenia as well as improve negative symptoms and cognitive impairment.

At present, the primary mechanism action of traditional antipsychotics is still to block the signaling of postsynaptic dopaminergic in the brain (Behr et al., 2000; Ichikawa et al., 2001; Muller-Spahn, 2002). Preclinical and clinical studies have clearly indicated that fronto-cortical dopamine system hypoactivity and striatal dopamine system hyperactivity associated with the occurrence of psychotics (McCutcheon et al., 2019; Rao et al., 2019). It is suggested that simultaneous blocking of dopamine D_2_ and 5-HT_2A_ receptors improves the efficacy of antipsychotic drugs in patients with schizophrenia and reduces the risk of extrapyramidal symptoms (EPS) (Andree et al., 1997; Kusumi et al., 2015). The conditioned avoidance response study is a well-established preclinical antipsychotic animal model (Wadenberg et al., 2000; Gao et al., 2015). Antipsychotic drugs can selectively suppress the conditioned avoidance response of rats. PK/PD (pharmacokinetics/pharmacodynamics) studies have suggested that the relationship between the suppression of conditioned avoidance response and dopamine D2 receptor occupancy of rats correlates well with the relationship between human clinical effects and dopamine D2 receptor occupancy (Wadenberg et al., 2001; Olsen et al., 2008). The present data indicate that NH300094 has good antagonism effects with D_2_ receptor (IC_50_ = 1.04 nM), and showed good efficacy in CAR study of rats. It predicts good clinic efficacy for NH300094. Apomorphine is a potent dopamine agonist, challenged with apomorphine can induce specific climbing behaviors in mice on subsequent occasions (Costall et al., 1978; Davis et al., 1986). Dopamine antagonists inhibit climbing behaviors of mice dose-dependently (Kafka and Corbett, 1996). NH300094 significantly inhibits apomorphine induced climbing, indicating strong dopamine antagonism effects. Taken together with the CAR results, the antipsychotic effects of NH300094 in animal studies correlate well with the *in vitro* antagonism activity of Dopamine D_2_ and D_3_ receptor.

Serotonin receptor, particularly the 5-HT_1A_ and 5-HT_2A_ receptors, are useful targets for the treatment of schizophrenia (Poyurovsky et al., 2003; Meltzer et al., 2012). In the prefrontal cortex, 5-HT_1A_ receptor and 5-HT_2A_ receptor are mostly expressed in pyramidal neurons and are involved in the regulation of excitatory and inhibitory transmission in these neurons, which accounts for the antipsychotic effects (Burnet et al., 1996; Amargos-Bosch et al., 2004; Santana et al., 2004). The DOI induced head-twitch behavior is a useful model for studying the activation of 5-HT_2A_ receptors in mice (Fantegrossi et al., 2010; Canal and Morgan, 2012). In 5-HT_2A_ receptor null-mutant mice, the DOI-induced head twitches are completely abolished (Gonzalez-Maeso et al., 2003). In our study, the ED_50_ value of NH300094 in mice DOI model was 0.006 mg/kg post p.o. administration. NH300094 dose-dependently inhibits DOI induced head-twitch behaviors, which is consistent with the *in vitro* data. The FLIPR assay shows that NH300094 is a potent 5-HT_2A_ receptor antagonist (IC_50_ = 0.47 nM). The density of 5-HT_1A_ receptor is increased in the brains of chronic schizophrenia patients, implying an important role of 5-HT_1A_ receptor in the pathogenesis of schizophrenia (Hashimoto et al., 1991; Millan, 2000). It is reported that antagonism of 5-HT_1A_ receptor can improve cognitive impairment in schizophrenia (Meltzer and Sumiyoshi, 2008). The stimulation of the 5-HT_1A_ receptor often interferes with memory-encoding mechanisms in brain, which leading to learning disabilities. However, antagonists of 5-HT_1A_ receptor can enhance cortical cholinergic/hippocampal and/or glutamatergic neurotransmission, which promoting certain types of memory (Ogren et al., 2008; Yamada et al., 2023). 5-HT_1A_ antagonists reversed the cognitive impairment induced by NMDA receptor antagonists or mACh receptor antagonists (Luttgen et al., 2005; Madjid et al., 2006). Lurasidone, a antipsychotics with potent 5-HT_1A_ antagonistic activity, has been shown to improve the learning and memory deficits induced by MK-801 in rats (Ishiyama et al., 2007; Horisawa et al., 2011) and to improve the cognitive impairment in schizophrenia in the clinic (Samalin et al., 2014; Meltzer et al., 2020). In our study, NH300094 but not risperidone shows good 5-HT_1A_ antagonistic activity in the c-AMP assay (NH300094 IC_50_ = 85.59 nM; risperidone IC_50_ = 4964.67 nM), and it has a very good *in vivo* efficacy in the NOR test in the scopolamine induced memory deficits model of mice. The minimal effects dose is lower than that of rivastigmine, a dementia disorders drug widely used for the treatment of Alzheimer’s disease (Marucci et al., 2021). Our data indicate that the 5-HT_1A_ antagonism activity of NH300094 might be one of the mechanisms of memory improvement effects.

Glutamatergic dysfunction is considered another mechanism of schizophrenia (Kruse and Bustillo, 2022). Studies have shown that the extracellular concentrations of dopamine and serotonin increase in the nucleus accumbens (NAC) and prefrontal cortex (PFC) after systemic administration of N-methyl-D-aspartic acid receptor (NMDA) antagonists such as MK-801, indicating that modulation of dopamine and serotonin receptors could potentially help restore the glutamatergic dysfunction in schizophrenia (Marcus et al., 2001; Lopez-Gil et al., 2007; DelArco et al., 2008). In rodents, MK-801, a NMDA non-competitive antagonist, induces complex behavioral syndromes that include locomotor hyperactivity, stereotypy, disruption of sensorimotor gating, and social deficit (Jentsch and Roth, 1999). Hyperlocomotion induced by acute MK-801 treatment in mice is a reliable and robust model for antipsychotic drugs testing (Ninan and Kulkarni, 1999). In our study, NH300094 effectively attenuated hyperlocomotion produced by MK-801, indicating its potential role in restoring the function of NMDA receptors in schizophrenia patients.

Cognitive impairment is one of the main obstacles to clinical and functional recovery in schizophrenia (Harvey et al., 2022). In patients with schizophrenia, D_3_ receptor levels are elevated in the limbic striatum, suggesting that D_3_ receptor antagonists might be effective in treating schizophrenia (Gurevich et al., 1997). The studies have suggested that dopamine D_3_ receptor antagonists could improve cognitive function of rats, which may be helpful in the clinical treatment of cognitive dysfunction associated with psychiatric disorders (Laszy et al., 2005; Watson et al., 2012). Huang et al. found that cariprazine could increase dopamine, norepinephrine, and serotonin efflux in both rat nucleus accumbens (NAC) and ventral hippocampus (HIP) via the antagonism of D_3_ activity (Huang et al., 2019). Selective dopamine D_3_ receptor antagonists (SB-277011A and SB-414796A) could enhance the extracellular levels of acetylcholine (ACh) in the rat medial prefrontal cortex (mPFC), which may be beneficial in the treatment of cognitive dysfunction (Lacroix et al., 2006). NH300094 has potent dual dopamine D_3_ receptor and 5-HT_1A_ receptor antagonism activity, which might account for its *in vivo* efficacy in improving cognitive function. Antagonism of 5-HT_1A_ receptor has been shown to ameliorate cognitive impairment in AD and schizophrenia (Meltzer and Sumiyoshi, 2008; Ogren et al., 2008; Shimizu et al., 2013). It is suggested that 5-HT_1A_ antagonists could improve cognitive function which mediated by postsynaptic 5-HT_1A_ receptor; However, full 5-HT_1A_ agonists impairs cognition by inhibiting the release of glutamate and acetylcholine in various regions of the brain (Jeltsch et al., 2004; Madjid et al., 2006). In addition, antagonism of dopamine D_3_ and 5-HT_1A_ receptor could increase the efficacy but decrease the side effects of antipsychotics. Unlike the typical motor side effects caused by D_2_ antagonists, the low brain abundance and peculiar distribution of D_3_ receptors become valuable targets for the development of drug (Maramai et al., 2016). KKHA-761, a potent dopamine D_3_ receptor antagonist, has antipsychotic activity with low risk of EPS (Park et al., 2005). The distinctive functional profile of clozapine may be related to its partial agonist activity against 5-HT_1A_ receptor (Millan., 2000). In our study, the unique profiles of dopamine D_3_ and 5-HT_1A_ receptor antagonism of NH300094 might account for its cognitive improvement activity and better safety profiles.

NH300094 is a novel antipsychotic with antagonist activity against 5-HT_2A_R > D_2L_R > D_3_R > 5-HT_1A_R receptors, showing powerful efficacy in positive, negative, and cognitive impairment animal models. *In vitro* mechanism studies showed that NH300094 could antagonize both dopamine receptor and 5-HT receptors, but its relative antagonistic potency against 5-HT_2A_ receptor was higher than that of dopamine receptors. Its strong agonistic activity against 5-HT_2A_ receptor may contribute to higher efficacy, overcome the limitations of current antipsychotics, and a better safety profile. Preclinical animal results showed that NH300094 was effective in multiple models, which is more extensive than the clinic drug Risperidone. The better safety margin of NH300094 may translate into a better clinical safety profile. In conclusion, NH300094 is a novel potent serotonin and dopamine receptors modulator, possessing potential for the treatment of schizophrenia with cognition disorder.

## Data Availability

The original contributions presented in the study are included in the article/Supplementary Material, further inquiries can be directed to the corresponding authors.
