# Supplementary material for: Assessing NH300094, a novel dopamine and serotonin receptor modulator with cognitive enhancement property for treating schizophrenia
Source: Front Pharmacol. 2024 Jan 24;15:1298061. doi: 10.3389/fphar.2024.1298061 (PMC10848157; doi:10.3389/fphar.2024.1298061)
Supplement: Supplementary file 1 [file Table1.DOCX]

Supplementary Material

**Materials and Methods**

**LC–MS/MS Instruments and Conditions**

Chromatographic separation was performed on an Agilent UHPLC system (Agilent Technologies, Palo Alto, CA) equipped with a binary pump, a sample manager, and a column manager. An ACQUITY UPLC® BEH C18 column (1.7µm, 2.1◊50 mm) was used and eluted by a binary mobile phase composed of acetonitrile (B) and 0.1% formic acid (v/v; A) following the gradient elution program (0-0.30 min: 20% B; 0.30-1.80 min: 20-100% B; 1.80-2.10 min: 100% B; 2.10-2.11 min: 100-20% B; 2.11-2.5 20% B). The column temperature was set at 40℃. The flow rate was 0.6 mL/min, and 1µL of the test solution was injected. An Applied Biosystems Triple Quad 5500 QTRAP® LC–MS/MS system (Toronto, Canada), including a hybrid triple quadrupole mass spectrometer, was equipped with a Turbo V^TM^ ion in the positive mode for data collection. The shared mass spectrometry parameters were 30 psi curtain gas, 50 psi nebulizer gas, 50 psi heater gas, medium collision gas, 500℃ ion spray temperature, and 5000 V ion spray voltage. The instrument control, data acquisition, and original data processing were performed using AB Sciex Analyst 1.7.1 software (Framingham, MA, USA)

**Figure Legends**

**Figure S1**. The detailed flow chart of the pharmacological study in vivo.

(A) The test flow chart of the MK-801-induced hyperactivity in mice.

(B) The test flow chart of the APO-induced climbing in mice.

(C) The test flow chart of the DOI-induced head twitch in mice.

(D) The test flow chart of the conditioned avoidance response test in rats.

(E) The test flow chart of the novel object recognition in mice.

(F) The test flow chart of the forced swimming test in mice.

(G) The test flow chart of the spontaneous locomotor activity test in mice.

(H) The test flow chart of the catalepsy test in mice.

**Table**

TABLE S1: In vitro functional profile of NH300094 and Risperidone

| Receptor | NH300094 | Risperidone | Positive control ^a^ |
| --- | --- | --- | --- |
| H_1_R, IC_50_(nM) | 126.32 | 432.85 | 7.11 |
| M_1_R, IC_50_(nM) | 3523.67 | >10000 | 2.17 |
| Alpha**_1A_**R, IC_50_(nM) | 28.48 | 48.90 | 131.62 |
| Alpha**_2A_**R, IC_50_(nM) | 313.63 | 745.37 | 21.84 |
| 5HT_2c_R, IC_50_(nM) | 3021.67 | 741.13 | 39.72 |
| 5HT_7_R, IC_50_(nM) | 5517.00 | 5.43 | 3.10 |

IC_50_, half maximal inhibitory concentration; EC_50_, half maximal effective concentration; R, receptor

^a^ positive control: Methiothepin (5HT_2c_R/5HT_7_R), Pyrilamine (H_1_R), Atropine (M_1_R), Prazosin (Alpha**_1A_**R), Yohimbine (Alpha**_2A_**R)
