# Supplementary figures and images for: Assessing NH300094, a novel dopamine and serotonin receptor modulator with cognitive enhancement property for treating schizophrenia
Source: Front Pharmacol. 2024 Jan 24;15:1298061. doi: 10.3389/fphar.2024.1298061 (PMC10848157; doi:10.3389/fphar.2024.1298061)

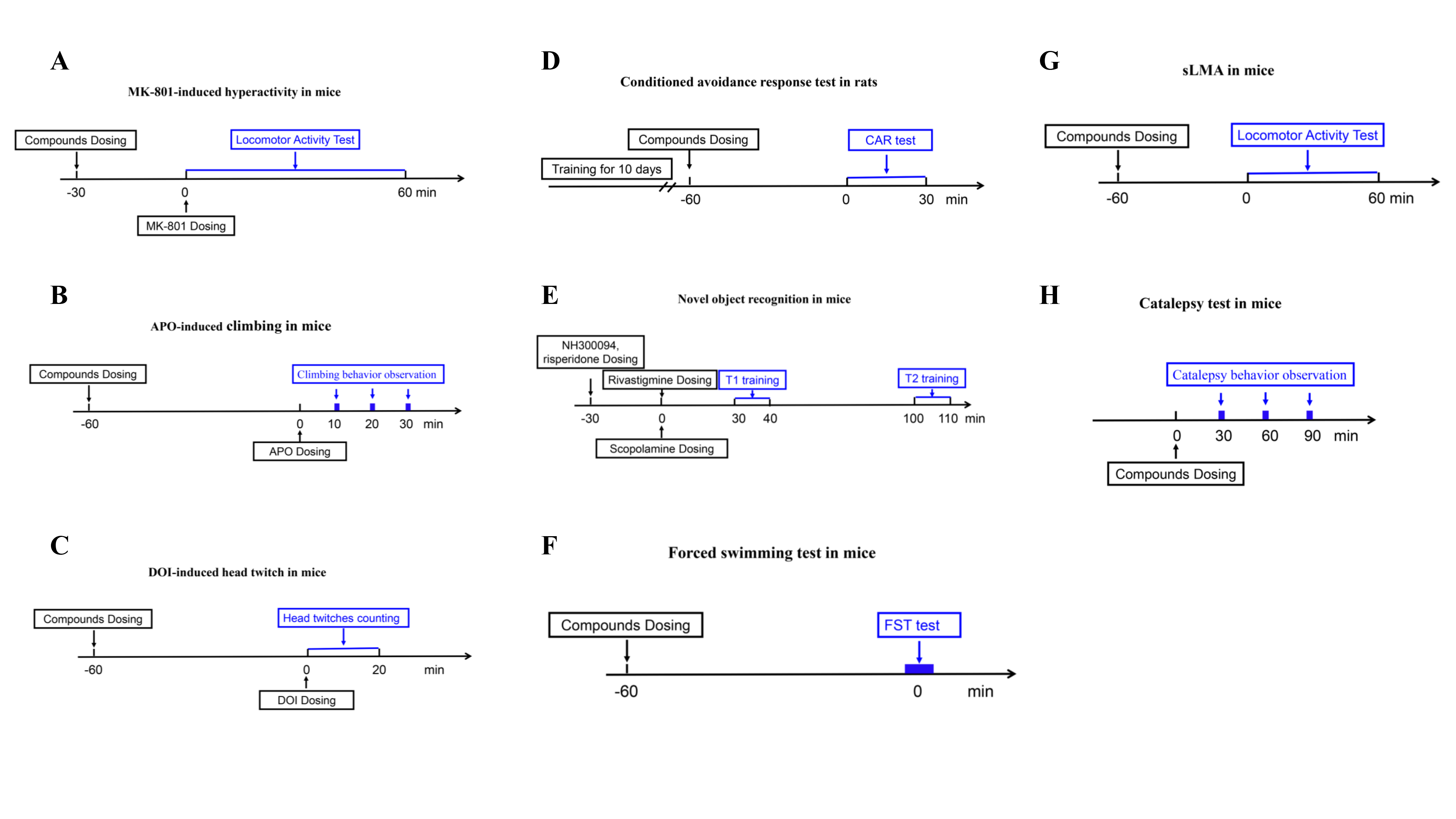

Supplement: Supplementary file 2 [file Image1.tif]
